# Effects of a 4–Week Detraining Period After 12 Weeks of Combined Training Using Different Weekly Frequencies on Health–Related Physical Fitness in Older Adults

**DOI:** 10.3390/ijerph21111433

**Published:** 2024-10-29

**Authors:** Lucas Betti Domingues, Vinícius Mallmann Schneider, Rodrigo Flores de Abreu, Leandro de Oliveira Carpes, Rodrigo Ferrari

**Affiliations:** 1 Postgraduate Program in Cardiology, School of Medicine, Universidade Federal do Rio Grande do Sul, Porto Alegre 90035-903, RS, Brazil; lucas.bdomingues@gmail.com (L.B.D.); vmallmannschneider@gmail.com (V.M.S.); rfabreu@hcpa.edu.br (R.F.d.A.); leandrocarpes.personal@gmail.com (L.d.O.C.); 2 Sports and Exercise Training Study Group, Clinical Research Center, Hospital de Clínicas de Porto Alegre, Porto Alegre 90470-260, RS, Brazil; 3 Postgraduate Program in Human Movement Sciences, School of Physical Education, Universidade Federal do Rio Grande do Sul, Porto Alegre 90040-060, RS, Brazil

**Keywords:** functional capacity, dose–response

## Abstract

Background: Detraining refers to the decline in physical fitness that occurs after the cessation of exercise, compromising the adaptations resulting from regular exercise training. To understand how long the benefits acquired from an exercise program can be maintained, the present study evaluated the detraining effects of a 4–week exercise cessation period in older adults who performed combined training at various weekly frequencies for 12 weeks. Methods: This randomized controlled trial assigned participants to one of two training programs: a combined training program twice a week (CT2) or four times a week (CT4) over a period of 12 weeks, followed by a four–week detraining period. The resistance training consisted of six bodyweight exercises, while the aerobic training involved either walking or running. Both the CT2 and CT4 groups completed the same total training volume and overload each week; the only difference was the number of training sessions per week. Assessments were conducted at baseline, after the training period, and after the detraining period, and included the 30–s and five–repetition chair–stand tests, isometric handgrip strength, body mass index (BMI), waist circumference, and waist–to–height ratio. Results: Thirty–one participants completed the study (CT2: 17 and CT4: 14). The groups presented similar attendance records during the training period (CT2: 96 ± 18% versus CT4: 94 ± 19%). After the 12–week training period, CT2 and CT4 improved lower limb strength, CT2 improved upper limb strength, and CT4 reduced waist circumference and waist–to–height ratio compared to baseline. After the 4–week detraining period, the lower limb strength remained improved in both groups (CT2: 4 ± 1 repetition; *p* < 0.001 and CT4: 4 ± 1 repetition; *p* < 0.001) when compared to the corresponding baseline values. The handgrip strength decreased in CT2 compared to post–training values. And the body composition benefit in CT4 was not sustained after detraining. Conclusions: The effects of 4 weeks of detraining after 12 weeks of training performed two or four times per week are similar on some but not all health-related physical fitness parameters.

## 1. Introduction

The regular practice of physical exercise is essential to mitigate the age-related declines of the neuromuscular system [1,2], resulting in improvements of physical function, balance, strength, quality of life, and the ability to perform activities related to daily living. [3]. Current guidelines recommend the combination of aerobic and resistance exercises (i.e., combined training) to elicit broad–spectrum benefits in aging populations. Aerobic exercise is the most widely acknowledged mode of exercise training for enhancing cardiorespiratory fitness. Resistance exercise represents a strategy for preventing and treating muscle weakness, offering benefits in terms of strength and muscle mass [4].

Different training variables—such as volume, intensity, and frequency—as well as the training status of participants can influence training adaptations. It is well documented that exercise programs with higher intensities and volumes lead to greater improvements in physical function for older adults [5,6]. The number of exercise sessions per week is another key factor that affects adaptations. While training frequency may influence some physical fitness parameters in older adults, different weekly frequencies do not uniformly impact all health–related parameters [7]. Studies on older men [7] and women [8] indicate that similar neuromuscular benefits can be achieved from training two to three times per week. However, in these studies, the groups did not undertake the same total amount of exercise weekly, highlighting the need for further research to clarify how varying training frequencies with the same total volume affect adaptations. Additionally, it is important to investigate how long the benefits of training can be maintained after different training frequencies.

To be efficacious, exercise programs must be conducted continuously and consistently. Discontinuation of established exercise routines is prevalent, occurring for various reasons, including injuries, diminished motivation, and vacation periods [9,10]. Detraining refers to the decline in physical fitness that occurs after the cessation of exercise, compromising the adaptations resulting from regular exercise training [11,12]. Consequently, elucidating the duration for which the benefits acquired from an exercise program can be maintained is crucial for achieving long–term objectives.

The data on the effects of detraining on health–related physical fitness parameters are inconclusive [12,13,14]. After a brief detraining period, some retention of maximum strength and power is observed, with these values remaining above baseline. However, in previously untrained individuals, short–term detraining periods of 3 to 4 weeks can lead to significant losses in muscle strength and power, especially among older adults [15,16]. These fitness parameters are critical for older adults due to their strong correlation with functionality and physical independence. Moreover, it is important to identify the minimum amount of exercise needed to maintain the adaptations gained during training, even after a period of detraining. This is particularly relevant for preserving physical function and minimizing conditioning loss, especially in those at higher risk of functional decline, such as older adults [17].

Considering the importance of studying the dose–response of exercise and the possibility of the exercise benefits to be lost partially or totally with detraining, the purpose of this study was to evaluate the detraining effects of a 4–week exercise cessation period on health–related physical fitness parameters in older adults who performed combined training twice or four times per week.

## 2. Materials and Methods

### 2.1. Study Design

This sub–study is part of an ongoing randomized controlled trial [18]. This exploratory analysis measured the detraining impacts of a 4–week exercise session period on muscular strength and body composition in older adults following 12 weeks of combined training performed twice or four times per week. The document was redacted following the Consolidated Standards of Reporting Trials (CONSORT) guidelines and its extension to non–pharmacological clinical trials [19,20].

### 2.2. Participants

Participants who took place in the study were aged between 50 and 80 years old, with a medical diagnosis of arterial hypertension or taking at least one antihypertensive medication, and not exercising regularly (two or more times a week) in the three months preceding the start of the study. Participants were excluded if they had musculoskeletal injuries or limitations that prevented them from carrying out the exercise program, a diagnosis of a cardiovascular disease such as acute myocardial infarction, stroke, angina, or heart failure class NYHA III and IV 24 months before taking part in the study, diseases that reduce life expectancy, such as cancer, kidney disease requiring hemodialysis, multiple sclerosis, Parkinson’s disease, BMI > 39.9 kg/m^2^, or a medical diagnosis of proliferative diabetic retinopathy, peripheral neuropathy, or other complications resulting from Diabetes Mellitus. Participants were recruited through telephone contact, posting on the social networks of the centers involved in the study, or by referral. Participation was voluntary and all the ethical, confidentiality, and data protection principles contained in the Declaration of Helsinki and the General Data Protection Act (NR 466/12) were adopted to preserve the identity of the participants.

### 2.3. Randomization and Allocation Concealment

The randomization list was generated by an independent researcher who did not take part in the recruiting process, evaluating, and monitoring the participants. Participants were only made aware of their allocation on the day they started training. The list was generated using the online software random.org (Randomness and Integrity Services Ltd., Dublin, Ireland). Stratified randomization was adopted in blocks of random sizes, and an allocation ratio of 1:1. The participants who were allocated to the intervention groups were stratified by sex (male; female), age (50–64 years; 65–80 years), and value of systolic blood pressure, assessed through 24 h of ambulatory monitoring, to ensure homogeneity in these prognostic factors between the groups. Age stratification was implemented to account for differences in outcomes, as age can significantly influence physiological adaptations and treatment effects.

The researchers responsible for the interventions did not have access to the results of the assessments, nor did the outcome assessors have access to the randomization list during the study period to ensure that the evaluations were not interfered with. The participants and the researchers responsible for the training could not be blinded to the experimental groups due to the nature of the interventions.

### 2.4. Preliminary Evaluations

During the run–in period, the study participants were submitted to a standardized anamnesis, a clinical examination including anthropometric assessments, resting electrocardiogram, and a cardiopulmonary exercise test. Those participants who revealed alterations in the resting electrocardiogram underwent an exercise electrocardiogram to confirm their eligibility for the study. Despite that, office blood pressure (BP) was obtained according to standardized guidelines [21].

Office BP and heart rate were measured after 20 min of rest, with the participant sitting quietly in a chair [21]. The participants were instructed to avoid physical exercises and alcohol ingestion 24 h before the exam. The measurements were taken using the validated automatic oscillometric device HBP-1100 monitor (OMRON Healthcare, Kyoto, Japan). Three measurements, 1–2 min apart, were performed in the arm with the highest initial systolic blood pressure value. The average of the two last measurements was considered the office blood pressure. The arm with the highest systolic blood pressure was consistently used as the reference for all measurements throughout the study.

The cardiopulmonary exercise test was performed on a treadmill to determine peak oxygen consumption. The protocol consisted of an initial 3.5 km/h speed with a 1% incline during the first 2 min. Subsequently, the speed and the degree of incline were increased by 0.4–0.6 km/h and 0.5–1.0% every 1 min, until the participants reached voluntary exhaustion. To confirm exhaustive effort, participants needed to meet at least one of the following criteria post-tests: (1) attaining volitional fatigue; (2) achieving a respiratory exchange ratio (RER) ≥1.1; or (3) reaching a heart rate (HR) ≥ 95% of estimated maximal HR through Tanaka’s Equation (=208 − 0.7 × age) [22]. Through visual inspection, two independent reviewers determined peak oxygen consumption (VO2peak) blindly. Expired gas was analyzed using a metabolic cart (Metalyzer 3B Cortex, Leipzig, Germany). A heart rate monitor (Polar H10, Finland) continuously monitored and recorded heart rate throughout the test. The incremental exercise test was conducted under the direct supervision of a licensed physician.

### 2.5. Outcome Assessments

To assess the effect of a 4–week detraining period after combined training performed twice or four times a week on health-related physical fitness, evaluations were performed before the intervention period (baseline), after the 12-week intervention (post–training), and a 4–week detraining period after the exercise completion (post–detraining). The outcomes included the chair–stand test and isometric handgrip test (primary outcomes), body mass, body mass index, waist circumference, and waist–to–height ratio (secondary outcomes).

Muscular strength was assessed using two tests: the chair–stand test for lower limbs and the isometric handgrip strength test for upper limbs. For the chair–stand test, a folding chair without arms and with a seat height of 43.2 cm was used. The participant began seated in the middle of the chair, with the back straight and feet approximately shoulder-width apart. The feet were placed on the floor at an angle slightly behind the knees. The arms were crossed at the wrists and held against the chest. At the signal “go”, the participant stood up with their body erect and straight, then returned to the initial seated position. The total number of stands completed correctly within 30 s, as well as the time taken to perform the first five repetitions, were recorded for analysis [23]. Isometric handgrip strength was measured in both arms using an analog hand dynamometer (Jamar Sammons Preston Rolyan, Bolingbrook, IL, USA). The participant sat upright with his or her forearm parallel to the ground and his or her elbow flexed at 90 degrees. He or she was instructed to perform a maximal squeeze for a sustained isometric effort lasting 5 s. Each hand underwent three attempts, with 1 min of rest between attempts.

Body composition was assessed using the body mass index calculated by the equation (body mass/height^2^) and the waist–to–height ratio (waist/height). Body mass and height were measured using an analog scale with a stadiometer (FILIZOLA, Brazil). Waist circumference was measured with an inelastic standardized measuring tape (Cescorf, Brazil) at the midpoint between the upper iliac crest and the lower costal rib on the horizontal plane.

### 2.6. Combined Training Program

Participants were randomized to a combined training group performed twice a week (CT2) or a combined training group performed four times a week (CT4) conducted on an outdoor athletics track (Porto Alegre, RS, Brazil). The CT2 and CT4 groups performed the same total training volume and overload per week (i.e., minutes of exercise per week, number of sets, number of repetitions, resistance exercises, intervals between sets and repetitions, aerobic exercise modality, and relative intensity of resistance and aerobic training), and only the number of training sessions per week differed. The training protocol lasted 12 weeks and a progression in training volume was planned, thus the training protocol was organized into two mesocycles (mesocycle 1: weeks 1–6; mesocycle 2: weeks 7–12). Combined training periodization is shown in Table 1.

Participants performed both resistance and aerobic training in sequence in the same session, starting with the bodyweight–based resistance exercises immediately followed by the aerobic exercise. Researchers and experienced supervised all training sessions. CT4 performed four combined training sessions a week composed of 10–15 min of bodyweight-based resistance exercises (1–4 sets of 10–15 repetitions in each exercise) followed by 20–25 min of aerobic exercise (walking or running). CT2 performed two combined training sessions per week composed of 20–30 min of resistance exercise (1–4 sets of 10–15 repetitions) followed by 40–50 min of aerobic exercise (walking or running).

The intensity of the training sessions was monitored using the rate of perceived exertion (RPE) using the Borg CR10 scale and remained the same throughout the study [24]. In bodyweight–based resistance exercises, the intensity was observed in five exercises (i.e., push-up, squat, inverted row, calf raise, and crunch). The target exercise intensity was ~50–60% of 1 RM and 60% VO2peak (4–6 A.U. of CR-10 Borg scale) for the bodyweight-based resistance and aerobic exercises, respectively. In addition, heart rate was monitored (Polar H10, Finland) continuously in the first and the last sessions of each mesocycle.

The bodyweight resistance exercises included push–ups performed on street workout bars, inverted rows using an overhead strap attached to a bar, calf raises on a step, squats, and abdominal exercises on floor mats. Adjustments to these exercises were made as needed. Common modifications included switching from bilateral to unilateral movements for lower limb exercises and changing joint angles during push–ups and inverted rows. Rate of Perceived Exertion (RPE) was assessed after the second repetition of each weight increment, allowing participants 8 to 10 s to evaluate and rate their muscular sensations. During aerobic exercises, RPE was also assessed to gauge the individual’s overall perception of muscle fatigue, shortness of breath, and physical stress.

### 2.7. Detraining Period

All participants were requested not to perform any type of structured physical exercise for 4 weeks after the post–training measurements. However, they were to maintain their regular routines, including incidental physical activities such as walking, commuting, or daily household tasks, as well as their habitual dietary patterns. Any significant changes in physical activity or diet during this period were to be reported to the study team to monitor compliance and ensure consistency in lifestyle factors and to maintain their regular habits, including daily physical activities and nutritional habits.

### 2.8. Statistical Analysis

Data were collected on standardized forms identified by subject number and trial ID, and data management was performed using an Excel spreadsheet. The normality assumption was assessed using the Shapiro–Wilk test and visual inspection of Q–Q plots. If the normality assumption was verified, the quantitative variables are presented as means ± standard deviation (or standard error) with 95% confidence intervals (CI95%). If the assumption was unmet, the variables are presented as medians and interquartile ranges. Categorical variables are presented as absolute and relative frequencies. The continuous variables were analyzed using the independent samples *t*–test, while the categorical variables were analyzed using the Chi–square test. For the analysis of the outcome variables, the Generalized Estimating Equations statistical procedure was used considering the group factor (CT2 and CT4), the time factor (pre–intervention, post–training, and detraining), and the interaction between group and time. Post–hoc comparisons were run using the sequential Bonferroni test to adjust for multiple testing. Effect sizes were calculated using Cohen’s d, and the interpretation of the effect size adopted was based on the following criteria: less than 0.50, small; 0.50–0.79, medium; and at least 0.80, large. The significant difference between detraining and either post–traning or baseline for each dependent variable was evaluated to analyze the impact of detraining within each group. A statistically significant difference was considered at *p* < 0.05. Statistical analyses were performed using SPSS v.19 software (IBM Corp., Armonk, NY, USA).

## 3. Results

**Figure 1:**
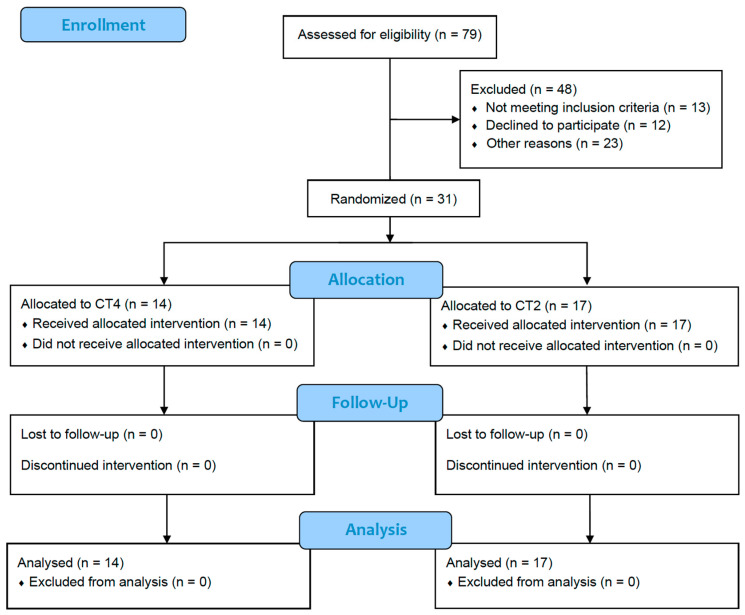
CONSORT flow diagram of the study participants. CT4, combined training intervention performed four times per week. CT2, combined training intervention performed twice a week.

Figure 1 shows the flow diagram of the study participants. Recruitment and follow-up assessments were conducted from September 2022 to December 2023. A total of 31 participants were randomly assigned to either CT4 (*n* = 14) or CT2 (*n* = 17). The attendance rate mean ± SD was 94 ± 19% for the 14 participants in CT4 group, while it was 96 ± 18% for the 17 participants of the CT2 group. No significant differences in attendance rates were observed between groups (*p* = 0.549). Additionally, no injuries related to the combined training programs were reported during the study follow–up period.

The baseline characteristics of the participants are described in Table 2. There were no baseline differences between the groups. Overall, participants presented elevated blood pressure and were classified as overweight or obese based on body mass index. Participants exhibited “Excellent” cardiorespiratory fitness according to the reference values for older adults provided by the American College of Sports Medicine [25].

**Figure 2:**
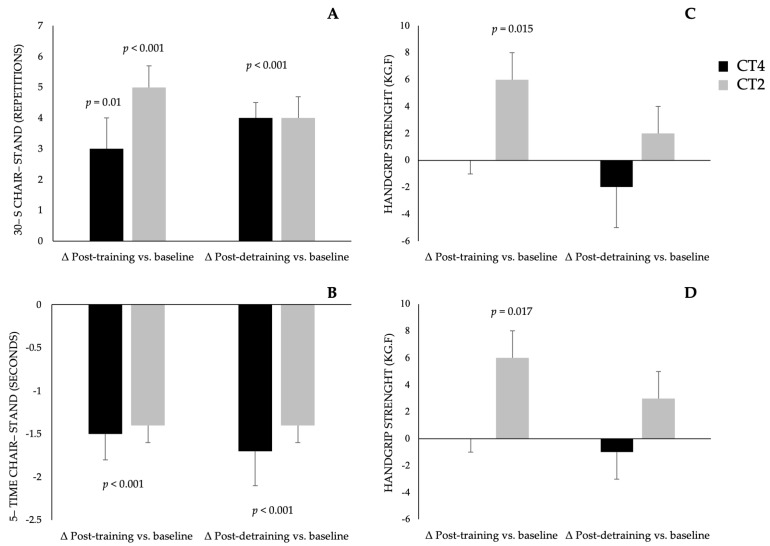
Difference in values between the post–training vs. Baseline and Post–detraining vs. baseline in the CT4 (combined training performed four times per week) and CT2 (combined training performed twice weekly). Data are presented as mean ± standard error. Comparisons were run using the Generalized Estimating Equations statistical procedure. *p* < 0.05 indicates a statistically significant difference in the time points. (**A**) 30–s chair–stand; (**B**) 5–time chair–stand; (**C**) Right arm grip strength; (**D**) Left arm grip strength.

The health–related physical fitness results are presented in Table 3 and Figure 2. After 12 weeks of training, both groups showed significant improvements in lower limb strength, as assessed by the 30–s chair–stand test and the five–time chair–stand test (*p* < 0.001), with these improvements maintained after the detraining period (*p* < 0.001). Upper limb strength, measured by the handgrip strength test, improved in the CT2 group after 12 weeks (*p* = 0.015) but was not sustained post–detraining. The inter–group effect size for the comparison between CT2 and CT4 was 0.0 for both the 30–s chair–stand test and the handgrip strength test. The intra–group effect size in CT4 was 1.0 for the 30–s chair-stand test and 0.1 for the handgrip strength test. In CT2, the effect size was 1.4 and 0.4 for lower and upper limb strength, respectively.

Regarding body composition, the CT4 group reduced waist circumference (*p* < 0.001) and waist–to–height ratio (*p* < 0.001) after the training period. However, these reductions were not sustained following the detraining period. The inter–group effect size between CT2 and CT4 was 0.0 for both BMI and waist–to–height ratio. The intra–group effect size for CT4 and CT2 was 0.0 for body mass, BMI, and waist–to–height ratio. For waist circumference, the effect size was 0.3 in CT4 and 0.1 in CT2.

## 4. Discussion

To the best of our knowledge, this is the first study evaluating the impact of detraining after combined training using different weekly frequencies on health–related physical fitness in older adults. The primary finding of the study was that improvements in lower limb muscular strength were maintained after a four–week period of detraining, regardless of the frequency of training. Considering that these benefits resulted from an exercise program that does not depend on specialized equipment, can be performed anywhere, and is both cost–effective and more accessible, these findings are clinically relevant, providing evidence that a bodyweight–based calisthenics combined with aerobic exercise program is effective to improve lower limb muscular strength, even when a short–term training cessation is necessary. However, upper limb strength improvements and waist circumference and waist–to–height ratio reductions were not sustained, indicating differential time–course effects of detraining in some physical fitness outcomes.

It is well–recognized that combined training effectively improves muscular strength in older adults [26,27]. Lower limb muscular strength, assessed by the 30–s chair–stand and 5–time chair–stand test, showed improvements after 12 weeks of combined training (~4 repetitions), regardless of the weekly frequency (i.e., twice and four times a week). The intra–group effect size was 1.0 for the CT4 group and 1.4 for the CT2 group, indicating a large impact on muscular strength adaptations. These gains were maintained after the four–week detraining period, representing a 20% increase compared to baseline values, with an inter–group effect size of 0.0, suggesting minimal differences between interventions.

Previous studies point out that the benefits of exercise on neuromuscular and physiological parameters can be maintained after shorter periods of detraining (i.e., four to eight weeks) [14,15]. In the present study, the improvement and maintenance of lower limb muscular strength after different weekly frequencies has important implications for exercise prescription in older adults. In contrast, upper limb strength, measured by the handgrip strength test, showed different results between the groups. Only the group that exercised twice a week showed significant improvements in handgrip strength, suggesting that the greater acute stimulus in the twice–weekly sessions in the CT2 group promoted better adaptations in handgrip strength. However, this benefit was not sustained after the detraining period. A possible explanation for the discrepancy between the detraining effects on upper and lower limbs is that forearm muscles are less engaged in daily living activities, resulting in a faster decrease in strength gains after detraining. In contrast, lower limb muscles, frequently used in daily activities such as walking, climbing stairs, and bending down, likely contributed to the maintenance of muscle strength in the lower limbs even after detraining [26,27].

Waist–to–height ratio and waist circumference are important parameters of central obesity, strongly associated with increased cardiovascular risk [28,29]. In this study, only the group that performed combined training four times per week reduced waist circumference and waist–to–height ratio after the training period. However, these improvements were not sustained after the detraining period. The inter–group effect size between CT2 and CT4 was 0.0 for both BMI and waist–to–height ratio, indicating a small effect, and 0.3 for body mass and waist circumference. Intra-group effect sizes for CT4 and CT2 were 0.0 for body mass, BMI, and waist–to–height ratio, while for waist circumference, they were 0.3 in CT4 and 0.1 in CT2, indicating a small effect. The group that exercised at a higher frequency may have had a higher energy expenditure compared to the group exercising twice per week [30]. However, the reversal of these gains after detraining underscores the importance of continuous training to maintain improvements in body composition, as even relatively short periods of detraining can significantly impact these adaptations. Future research should further investigate the effects of detraining on other body composition parameters to support these findings.

Some limitations should be considered when interpreting the results, such as the short detraining period, which does not allow for an assessment of the effects of longer interruptions on functional capacity, and the evaluation of older individuals, which limits the comparison of data with data gathered from younger populations. Furthermore, the use of specific weekly frequencies (i.e., two and four sessions per week) and the assessment of physically inactive individuals limit the generalizability of the findings to those who train with different weekly frequencies and to physically active individuals. Despite these limitations, this study employed a pragmatic intervention approach, requiring minimal structure for the implementation of the exercise protocol. The evaluations used are both low-cost and highly reproducible, enhancing the feasibility of the protocol. These characteristics make it easily adaptable to various real-life settings, such as health units, outdoor gyms, public squares, and other accessible locations. This provides relevant data for professionals to prescribe exercise in real–life environments. Moreover, it is important to note that the total training volume was equalized in this study, addressing a common confounding factor in research comparing different weekly frequencies. The investigators who allocated and analyzed the data were blinded to enhance transparency and avoid measurement bias.

## 5. Conclusions

The improvement of lower limb muscular strength resulting from a 12–week pragmatic combined training program performed two or four times per week were similarly sustained after a four–week of exercise cessation in older adults. Additionally, the post-training improvements in upper limb strength (CT2) and body composition (CT4) were not sustained after detraining. Taken together, the effects of 4 weeks of detraining after 12 weeks of training performed two or four times per week are similar on some but not all health–related physical fitness parameters.

## Figures and Tables

**Table 1:** Combined resistance and aerobic training periodization.

Mesocycle 1			Mesocycle 2	
Modalities	Volume	Intensity	Volume	Intensity
Resistance exercises	Sets × Repetitions	RPE	Sets × Repetitions	RPE
Push-up ^1^	2 × 10–12	4–5	3 × 10–12	5–6
Squat ^1^	3 × 12–15	4–5	4 × 12–15	5–6
Unilateral balance ^1^	1 × 30″	–	1 × 45″	–
Inverted row ^2^	2 × 10–12	4–5	3 × 10–12	5–6
Calf raise ^2^	2 × 12–15	4–5	3 × 18–20	5–6
Crunch ^2^	2 × 15	4–5	3 × 20	5–6
Aerobic exercise	Minutes per week	RPE	Minutes per week	RPE
Walking/Running ^3^	80′	5–6	100′	5–6

Combined training periodization of combined training performed twice (CT2) and four times (CT4) per week. ^1^ Resistance exercises performed on days 1 and 3 in the CT4 group; ^2^ Resistance exercises performed on days 2 and 4 in the CT4 group; ^3^ Minutes per week of aerobic training was fractionated accordingly in the weekly training group.

**Table 2:** Clinical and Sociodemographic Characteristics of the Study Participants.

Variables	CT4 (*n* = 14)	CT2 (*n* = 17)	*p* Value
Sex, *n* (%)			0.934
Male	7 (50)	8 (47.1)	
Female	7 (50)	9 (52.9)	
Age, years	66 ± 5	66 ± 8	0.924
Anthropometry			
Body mass (kg)	75.1 ± 11.8	77.5 ± 15.7	0.281
Height (m)	1.63 ± 0.1	165 ± 0.1	0.288
BMI (kg/m^2^)	28.1 ± 3	28.3 ± 4.5	0.629
Waist circumference (cm)	92.6 ± 10.8	97.9 ± 12.8	0.450
BMI classification, *n* (%)			0.066
Eutrophic	2 (14.3)	5 (29.4)	
Overweight	10 (71.4)	5 (29.4)	
Obesity	2 (14.3)	7 (41.2)	
Cardiorespiratory fitness			
Peak VO2 (mL.kg.min^−1^)	24.8 ± 4.9	28.6 ± 5.9	0.081
HR VT1 (bpm)	100 ± 15	98 ± 11	0.640
HR VT2 (bpm)	126 ± 14	125 ± 14	0.941
Hemodynamics			
Systolic BP (mmHg)	129 ± 13	128 ± 16	0.969
Diastolic BP (mmHg)	77 ± 11	73 ± 9	0.283
Heart rate (bpm)	73 ± 13	66 ± 8	0.078
RPP (mmHg.bpm)	9483 ± 2100	8490 ± 1443	0.149
Antihypertensive drugs, *n* (%)			
ACEI	8 (57.1)	4 (23.5)	0.225
ARB	6 (42.9)	9 (52.9)	0.390
Diuretics	7 (50)	10 (58.8)	0.823
β blockers	2 (14.3)	4 (23.5)	0.736
CCB	2 (14.3)	6 (35.6)	0.328
Hypoglycemic drugs, *n* (%)	2 (14.3)	1 (5.9)	0.747
Hypolipidemic drugs, *n* (%)	10 (71.4)	13 (76.5)	0.736

Quantitative variables are presented in mean ± standard deviation and categorical variables in absolute (relative) frequency. CT4, combined training intervention performed four times per week. CT2, combined training intervention performed twice a week. BMI, body mass index. VT, ventilatory threshold. HR, heart rate. BP, blood pressure. RPP, rate–pressure product. ACEI, angiotensin II receptor blockers. CCB, calcium channel blockers.

**Table 3:** Functional capacity and body composition measurements following 12 weeks of combined training performed twice or four times a week and after 4-week detraining period.

	CT4 (*n* = 14)			CT2 (*n* = 17)			*p* Value		
Variables	Baseline	Post–Training	Post–Detraining	Baseline	Post–Training	Post–Detraining	Interaction	Time	Group
Functional capacity									
30–s chair-stand (repetitions)	20 (17–23)	23 (21–25) ^1^	24 (22–27) ^2^	19 (18–21)	24 (22–26) ^1^	23 (22–25) ^2^	0.160	<0.001	0.934
5–time chair-stand (seconds)	8.5 (7.5–9.5)	6.9 (6.2–7.7) ^1^	6.8 (5.9–7.6) ^2^	8.2 (7.6–8.8)	6.8 (6.3–7.3) ^1^	6.8 (6.4–7.2) ^2^	0.580	<0.001	0.777
Right arm grip strength (kg·F)	33 (27–40)	33 (28–39)	31 (26–37)	32 (28–36)	38 (34–43) ^1^	34 (30–40) ^3^	0.042	0.004	0.507
Left arm grip strength (kg·F)	32 (26–38)	32 (27–38)	31 (24–38)	29 (25–33)	36 (31–41) ^1^	32 (27–37) ^3^	0.068	0.014	0.852
Body composition									
Body mass (kg)	75 (69–81)	75 (68–81)	75 (69–80)	78 (70–85)	78 (70–86)	78 (70–86)	0.747	0.968	0.572
BMI (kg·m^2^)	28.1 (26.7–29.5)	28 (26.5–29.5)	28 (26.5–29.5)	28.3 (26.2–30.4)	28.4 (26.1–30.6)	28.4 (26.1–30.6)	0.894	0.952	0.816
Waist circumference (cm)	96.6 (91.2–101.9)	92.7 (87.9–97.6) ^1^	95.4 (91.1–99.8) ^3^	97.9 (91.9–103.7)	96.6 (89.8–103.4)	97.4 (90.4–104.4)	0.188	0.001	0.568
Waist–to–height ratio (A.U.)	0.59 (0.56–0.63)	0.56 (0.54–0.60) ^1^	0.59 (0.55–0.62) ^3^	0.59 (0.56–0.63)	0.59 (0.55–0.62)	0.59 (0.55–0.63)	0.170	0.002	0.821

Data are shown in mean (95% confidence interval). BMI, body mass index. CT4, combined training performed four times per week. CT2, combined training performed twice weekly. ^1^ Denotes statistical difference between Baseline and Post–training. ^2^ Denotes statistical difference between Baseline and Post–detraining. ^3^ Denotes statistical difference between post–training week and post–detraining.

## Data Availability

The data presented in this study are available on request from the corresponding author.
